# Dataset on the mean, standard deviation, broad-sense heritability and stability of wheat quality bred in three different ways and grown under organic and low-input conventional systems

**DOI:** 10.1016/j.dib.2016.04.065

**Published:** 2016-05-04

**Authors:** Marianna Rakszegi, Franziska Löschenberger, Jürg Hiltbrunner, Gyula Vida, Péter Mikó

**Affiliations:** a Agricultural Institute, Centre for Agricultural Research, Hungarian Academy of Sciences, Brunszvik u. 2, 2462 Martonvásár, Hungary; b Saatzucht Donau GmbH & Co KG, Saatzuchtstrasse 11, 2301 Probstdorf, Austria; c Agroscope, Institut für Pflanzenbauwissenschaften IPB, Reckenholzstrasse 191, 8046 Zürich, Switzerland

## Abstract

An assessment was previously made of the effects of organic and low-input field management systems on the physical, grain compositional and processing quality of wheat and on the performance of varieties developed using different breeding methods (“Comparison of quality parameters of wheat varieties with different breeding origin under organic and low-input conventional conditions” [1]). Here, accompanying data are provided on the performance and stability analysis of the genotypes using the coefficient of variation and the ‘ranking’ and ‘which-won-where’ plots of GGE biplot analysis for the most important quality traits. Broad-sense heritability was also evaluated and is given for the most important physical and quality properties of the seed in organic and low-input management systems, while mean values and standard deviation of the studied properties are presented separately for organic and low-input fields.

**Specifications table**

**Table:** 

Subject area	Biology
More specific subject area	Agriculture, organic farming, wheat breeding
Type of data	Tables and Figures
How data was acquired	Foss Tecator 1241, Perten Falling Number system 1500, Kjeltec 1035 Analyser, Glutomatic 2200, SediCom System, Marvin System, Perten SKCS 4100, Brabender Farinograph
Data format	Analysed
Experimental factors	Seed was milled on a Chopin CD1 laboratory mill
Experimental features	Physical, grain compositional and processing quality properties of the wheat were measured with standard methods
Data source location	Martonvasar, Hungary
Data accessibility	Data are provided in the paper

**Value of the data**

• This data set could help to select stable varieties for organic or low-input farming purposes.
• As the data originate from and compare organic and low-input conventional systems this makes it possible to compare them with organic contra high-input conventional systems.
• Although the varieties compared included the product of organic, conventional and combined (BFOA) breeding, conventional varieties were still dominant, which highlights the need for development in the field of organic ‘breeding’ and farming.
• The practical usefulness of the stability analysis could be even better if a more robust variety set and more growing sites were included in the analysis.

## 1 Data

Datas on the stability of thirty-seven wheat genotypes based on their physical, grain compositional and processing quality traits are presented using the coefficient of variation and GGE biplot analysis. The mean, standard deviation and broad-sense heritability values of the most important traits were also calculated for organic and low-input systems distinguishing the varieties with different breeding origin as well.

## 2 Experimental design, materials and methods

### 2.1 Plant material

Thirty-seven winter wheat varieties and breeding lines were sown at organic and conventional low input sites (hereafter’low input’) in two different countries (Austria, Hungary) in 2011, 2012 and 2013. The varieties originated from 5 different countries (Austria, France, Germany, Hungary and Switzerland) and were bred in three different ways [2], nine on certified organic fields (Donnato, Aszita, Wiwa, Scaro, Butaro, Jularo, Sandomir, Gulliver, Karachow), 20 on conventional fields (Mv Emese, Mv Béres, Mv Kolo, Mv Kolompos, Mv Tallér, Lukullus, Arnold, Capo, Midas, Claro, Lorenzo, Suretta, Titlis, Montdor, CH111-14426, CH111-14663, CH111-14631, Folklor, Renan, Flamenco), and eight (Blasius, Peppino, Pireneo, Stefanus, Bitop, Tobias, Hendrix, Skerzzo) using a combined method (breeding for organic agriculture-BFOA) including selection under conventional conditions in early generations (usually up to F5) followed by selection in late generations on certified organic farms [3]. Detailed information on the origin and agronomical properties of the varieties were published by Mikó et al. [4].

### 2.2 Plant growing conditions

Between 2011 and 2013, 37 bread wheat varieties were sown in Austria (A) and Hungary (H) using a similar randomised complete block experimental design with 3 replicated blocks under organic (O) and low input (LI) growing conditions. In both countries the O and LI sites were located on neighboring fields and the experiments were planted close to each other (<1080 m) to minimize the confounding effects of differences in soil and climatic conditions. Low-input systems could be characterized by a reduced level of mineral fertilizer, green manure, tillage and seed chemical treatment compared to high-input conventional farming systems. Furthermore, herbicides, insecticides and artificial fertilizers were used in the low input fields when necessary, but no fungicides. There was a serious *Tilletia caries* contamination in 2013 at organic sites in both countries, so fewer varieties and fewer quality parameters could be measured. In the low input fields, nitrogen was supplied using mineral fertilizers according to local practice, while only previous crops (mainly legumes) provided nutrient supplies at the organic locations (Table 1). Weed pressure was very low at the organic sites in both countries in all the years. The weather conditions differed greatly not only between the years but also between the countries. After the moderately dry first season of 2010/2011, the year 2012 saw an extreme drought, which was followed by an average season in 2013. In most cases, the Hungarian locations received less precipitation and were warmer than the Austrian ones.

### 2.3 Assessment of quality traits

#### 2.3.1 Physical properties

The test weight (kg/100 l) of the grain was measured using a Foss Tecator 1241 instrument (MSZ 6367/4-86). Thousand-kernel weight (TKW) was determined with the Marvin System according to the standard method MSZ 6367/4-86 (1986). A Perten SKCS 4100 instrument was used to measure the hardness of the kernels (AACC Method 55-31).

#### 2.3.2 Milling

Grain sample weighing 700 g from each of the 3 field replications were milled separately to flour using a Chopin CD1 Laboratory Mill after conditioning the grain to 15.5% moisture content. Wholemeal samples were produced from the same samples with a Perten 3100 Laboratory Mill.

#### 2.3.3 Grain composition

Crude protein content was analysed in duplicate with the Kjeldahl method, which is consistent with ICC method 105/2, using the Kjeltec 1035 Analyzer. Gluten content and gluten index (GI) were determined using a Glutomatic 2200 instrument (ICC 137/1, 155), and gluten spread according to the Hungarian standard MSZ 6369/5-87 (1987). This parameter provides information about the proteolytic activity of the samples by monitoring changes in the diameter of a gluten ball after 1 hour at room temperature. The starch content of the grain was measured with a Foss Tecator 1241 instrument. Basic grain compositional parameters were also estimated with the Near Infrared Spectroscopy (NIR) method (ICC 202 and ICC 159) using Foss Tecator 1241 and Perten Inframatic 8611 instruments for grain and flour, respectively.

#### 2.3.4 Breadmaking quality characters

A Brabender Farinograph (ICC 115/1) was used to determine flour water absorption, development time, stability and dough softening. The Zeleny sedimentation test was carried out according to standard ICC 116/1 and the falling number was measured with a Perten Falling Number System 1500 (AACC56-81B).

**Fig. 1:**
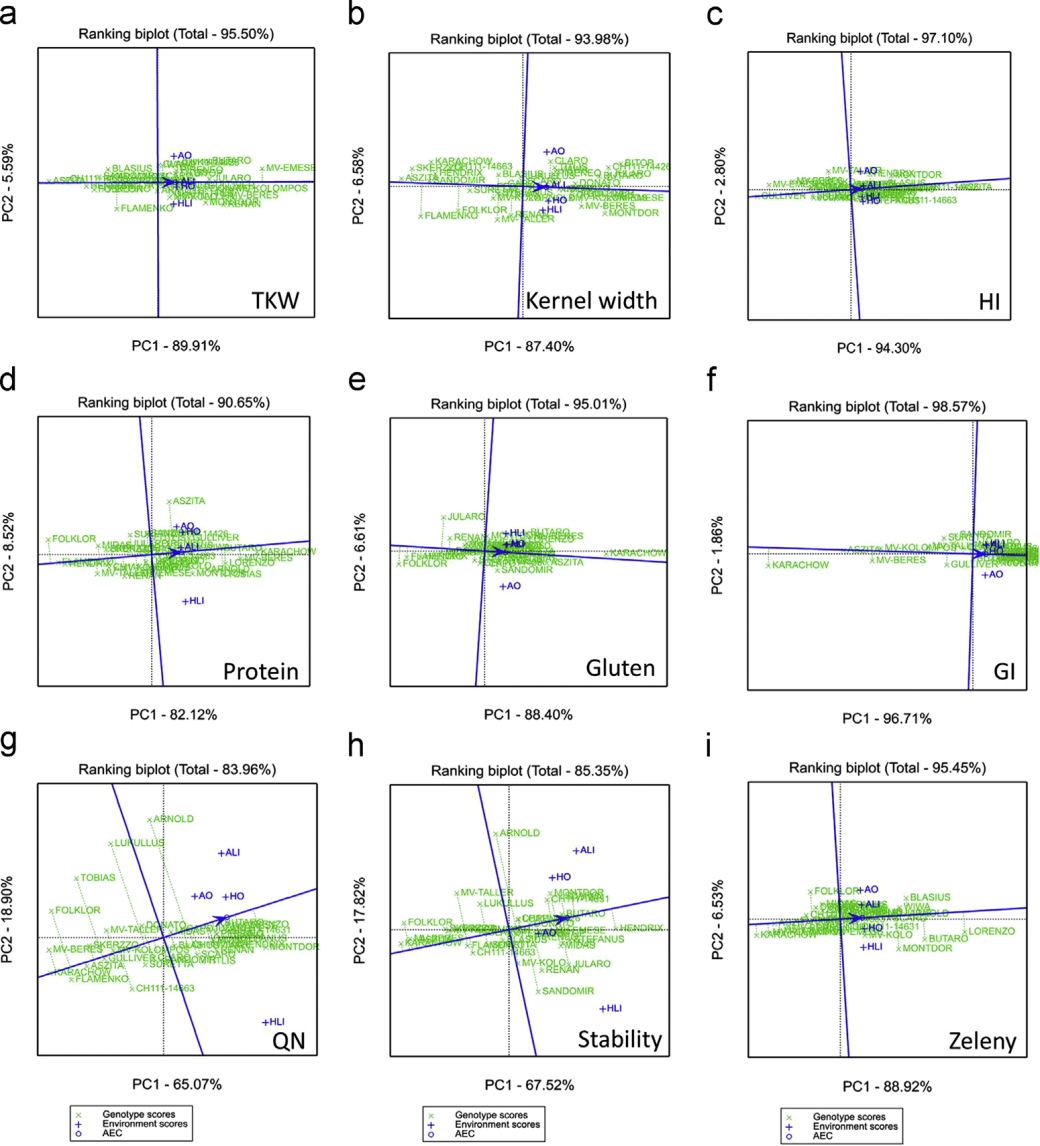
Stability and mean performance of the genotypes using the ranking plot of the GGE biplot for the traits: (a) thousand-kernel weight: TKW; (b) kernel width; (c) hardness index: HI; (d) protein content; (e) gluten content; (f) gluten index: GI; (g) Farinograph quality number: QN; (h) dough stability: stability; and (i) Zeleny sedimentation.

**Fig. 2:**
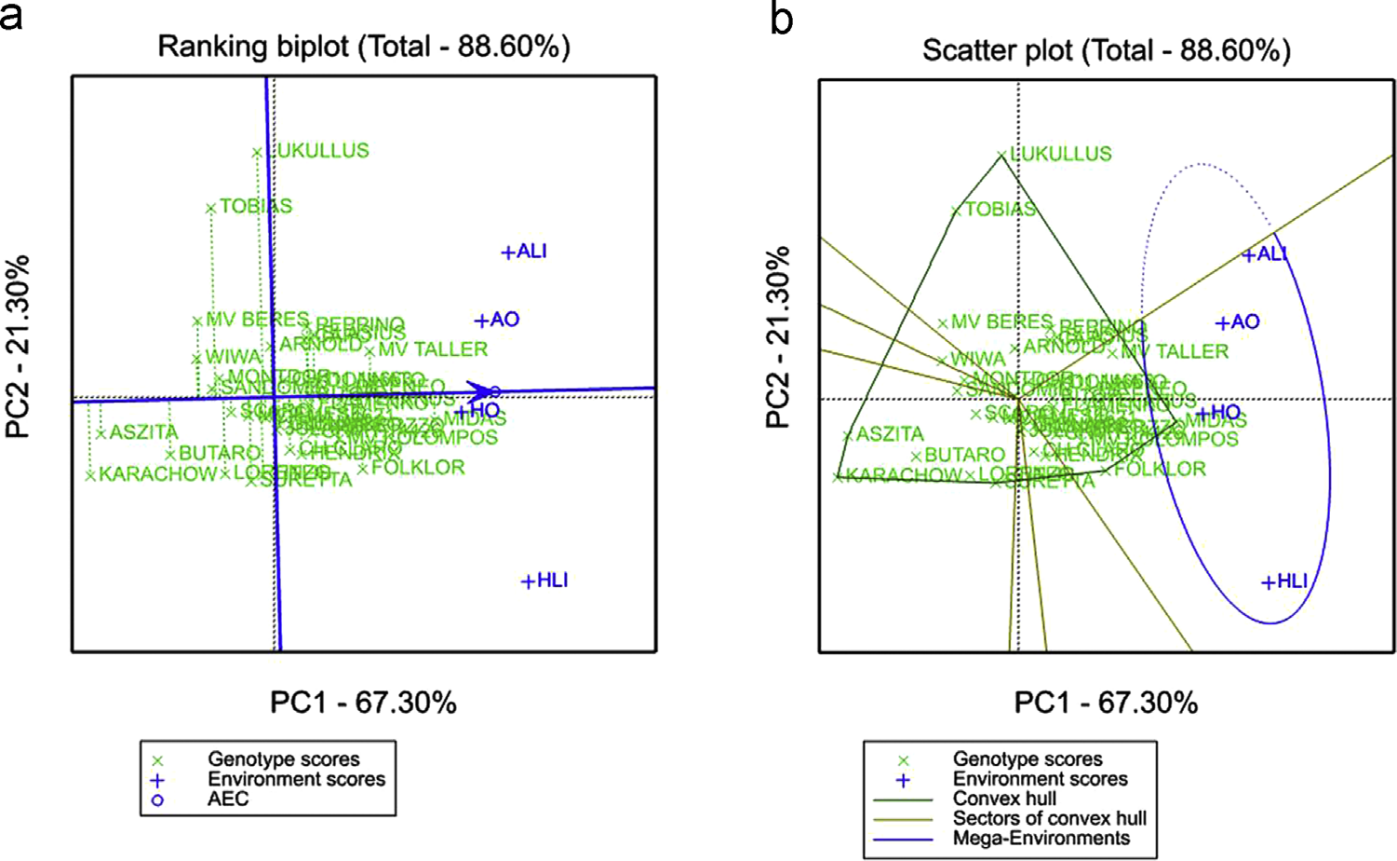
Ranking plot (a) and ‘which-won-where’ and (b) view of the GGE biplot for grain yield.

**Fig. 3:**
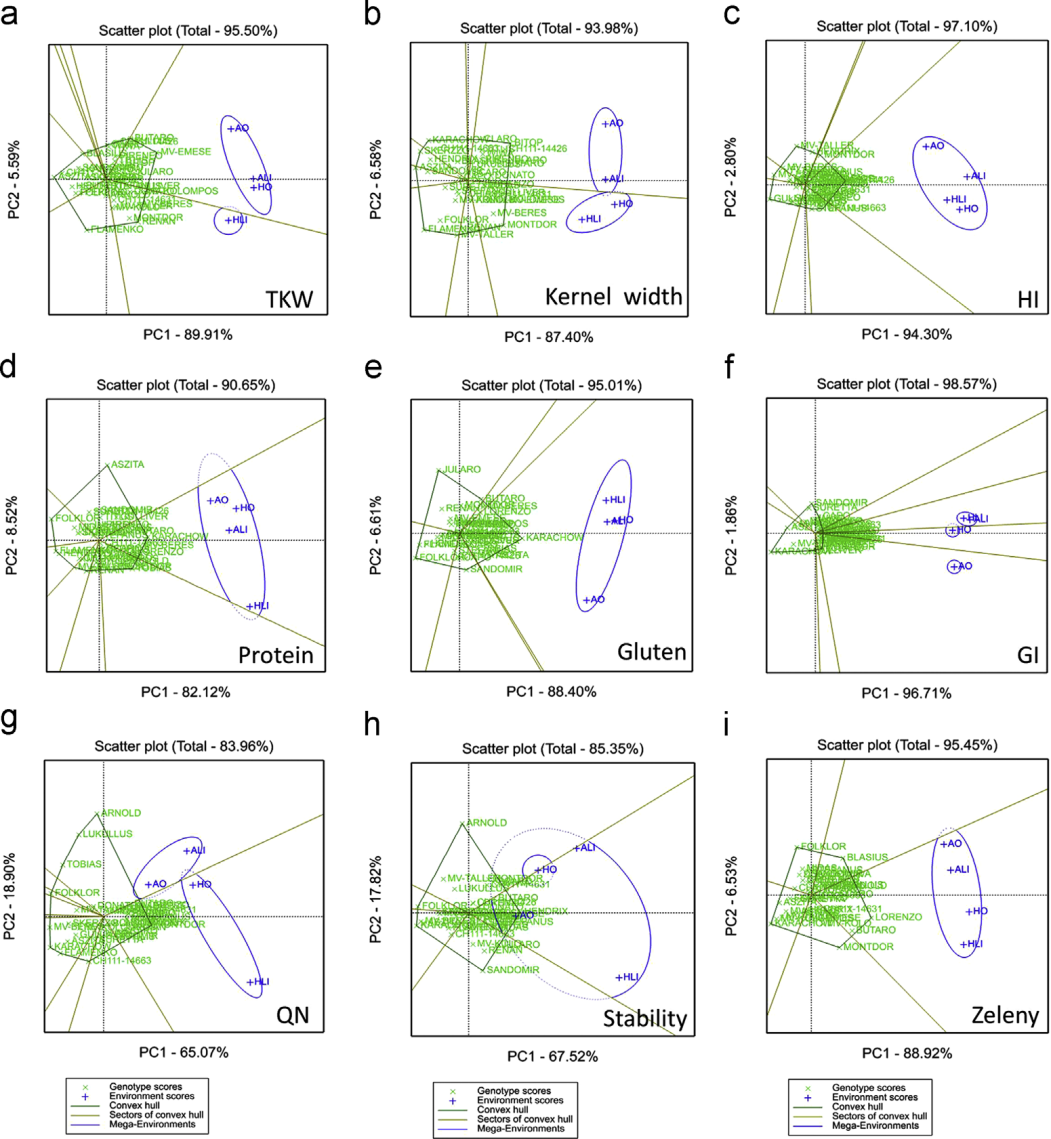
The ‘which-won-where’ view of the GGE biplot to show which genotype performed best in which environments ((a) thousand kernel weight: TKW; (b) kernel width; (c) hardness index: HI; (d) protein content; (e) gluten content; (f) gluten index: GI; (g) Farinograph quality number: QN; (h) dough stability: stability; and (i) Zeleny sedimentation).

These datas were evaluated in Table 2, Table 3, Table 4, Table 5 and Fig. 1, Fig. 2, Fig. 3.

### 2.4 Statistical analysis

Repeatability (broad-sense heritability) was calculated as the ratio of genotypic to phenotypic variance (Table 2) as described by Rakszegi et al. [1].

One-way ANOVA and Tukey׳s post hoc test were carried out using SPSS 16.0 software (SPSS Inc., Chicago, IL, USA) (Table 3, Table 4).

The coefficient of variation (CV), expressed as the ratio of the standard deviation and the mean, is a measure of the data variability. Low values of CV mean that the data has less variability and high stability (Table 5).

GGE biplot analysis was carried out using GenStat 17.0 software (VSN International Ltd., Hemel Hempstead, UK) [5]. The GGE biplot illustrates the genotype plus genotype-by-environment variation using scores from principal component analysis, but without environmental effects. The Ranking biplot (average-environment coordination (AEC) view of the GGE biplot) can be used to examine the performance of all genotypes within a specific environment. In the plot, the best performing and most stable genotypes are those whose projections onto the biplot axis are closest to the environment. The single arrow on the AEC abscissa points to higher mean values of a given trait. The distance from the AEC ordinate indicates greater variability (poorer stability) in both directions. The “which-won-where” function of the GGE biplot is an extended use of the ‘pair-wise comparison’ function and shows which genotype performed the best in which environment. Genotypes located on the vertices of the polygon performed either the best or the poorest in one or more environments present in the same sector (Fig. 1, Fig. 2, Fig. 3).

## Figures and Tables

**Table 1:** Main growing conditions and management parameters of organic and low input fields at trial locations in Hungary (H) and Austria (A) in three growing seasons in the winter bread wheat ring test.

**Growing conditions**			**Trial location: country×management system**			
**HO**	**HLI**	**AO**	**ALI**
**Location**	**Geographical coordinates**		47.3N, 18.8E		48.2N, 16.6E	
**Altitude**		115 m		150 m	
			
Weather conditions*	Total precipitation (mm)	2010/2011	248.7		367.6	
2011/2012	217.2		207.5	
2012/2013	387.5		485.3	
Average temperature (°C)	2010/2011	8.2		7.2	
2011/2012	8.4		7.8	
2012/2013	8.0		7.1	
				
**Average soil parameters**	Soil type		Chernozem	Chernozem	Chernozem	Chernozem
pH (KCl)		7.25	7.25	7.4	7.4
Humus (m/m %)		2.6	2.8	3.0	3.0
P_2_O_5_ (mg/kg)		440	210	144	144
K_2_O (mg/kg)		245	210	299	299
Yearly average of N input through NPK combined fertilizer (active ingredient, kg/ha)		0	120	0	126
					
**Growing parameters**	Previous crop	2010/2011	Sunflower	Maize	*Lathyrus* sp.	Sugar beet
2011/2012	Alfalfa	Oilseed rape	Field pea	Field pea
2012/2013	Field pea	Oilseed radish	Lentils/bitter-cress	Sugar beet
Sowing density		450 seeds/m^2^		350 seeds/m^2^	
Growing period (days)	2010/2011	272	271	264	268
2011/2012	260	260	275	262
2012/2013	271	271	274	273

* Parameters refer only to the growing period (basically between October and July).

**Table 2:** Broad-sense heritability (*h*^2^), mean values with standard deviations (SD), variance components estimate and their standard errors (±SE) for genotype (G), genotype×environment (G×E) interaction and error variance for grain yield, thousand-kernel weight, gluten spread and gluten index (2011–2013, Austria and Hungary, organic and low input sites).

**Trait (unit)**	**Organic management**									**Low input conventional management**								
Mean	SD	G		GxE		Error		***h***^**2**^	Mean	SD	G		GxE		Error		***h***^**2**^
***σ***^**2**^_**G**_	**±SE**	***σ***^**2**^_**G×E**_	**±SE**	***σ***^**2**^_**e**_	**±SE**	***σ***^**2**^_**G**_	**±SE**	***σ***^**2**^_**G×E**_	**±SE**	***σ***^**2**^_**e**_	**±SE**
**Grain yield (t/ha)**	4.38	1.71	0.07**	0.02	0.07***	0.02	0.25	0.02	0.85	4.61	2.08	0.08*	0.03	0.17***	0.03	0.41	0.03	0.70
**Thousand kernel weight (g)**	40.40	4.46	7.68***	1.99	1.64***	0.43	3.24***	0.32	0.96	41.79	5.35	7.95***	1.99	1.99***	0.35	1.45***	0.15	0.96
**Gluten spread (mm)**	4.81	2.99	6.23***	1.58	0.93***	0.19	0.84***	0.09	0.97	4.91	3.09	4.95***	1.31	2.63***	0.37	0.82***	0.09	0.92
**Gluten index**	90.23	12.34	126.83***	30.72	15.55***	2.72	8.25***	0.88	0.98	90.83	12.82	158.64***	37.64	15.27***	2.01	5.00***	0.53	0.98

*σ*^2^_G,_*σ*^2^_G×E,_*σ*^2^_e_=genotypic, genotype×environment interaction and residual variance components, respectively.

* Significant at the 0.05 probability levels, respectively.

** Significant at the 0.01 probability levels, respectively.

*** Significant at the 0.001 probability levels, respectively.

**Table 3:** Mean values of 37 genotypes for two countries and two management systems (GS: growing site, TW: test weight, TKW: thousand kernel weight, KW: kernel width, HI: hardness index, GI: gluten index, Wabs: water absorption, QN: quality number, ORG: organic variety, CONV: conventional variety, BFOA (breeding for organic agriculture): variety developed by a combined method, A: Austria, H: Hungary, O: organic site, LI: low input site).

Breeding	Genotype	GS	Yield	TW	TKW	KW	HI	Protein	Gluten	GI	Zeleny	Wabs	Stability	QN
			(t/ha)	(kg/100 l)	(g/1000)	(mm)		(%)	(%)		(ml)	(%)	(min)	
ORG	ASZITA	ALI	4.68	82.50	34.43	3.05	72.39	15.40	42.10	60.22	29.00	64.90	3.90	57.30
		AO	3.86	81.40	33.81	3.07	70.69	16.10	42.60	58.06	28.00	66.30	6.00	65.90
		HLI	3.95	83.40	35.41	3.10	71.93	14.40	42.13	62.68	30.00	67.10	4.90	62.40
		HO	4.30	79.80	34.32	3.02	75.86	15.70	43.95	67.27	30.00	69.30	7.40	72.80
														
ORG	BUTARO	ALI	5.20	83.70	44.93	3.35	54.02	16.40	42.04	86.60	43.00	68.50	14.00	88.60
		AO	3.83	84.10	45.22	3.35	56.62	16.00	35.18	90.30	39.00	72.70	6.10	75.70
		HLI	4.47	84.90	43.35	3.31	58.32	15.10	39.33	91.28	43.00	68.90	13.30	85.50
		HO	4.68	81.10	43.12	3.33	55.21	15.80	41.76	91.55	47.00	71.80	14.50	87.90
														
ORG	DONNATO	ALI	6.08	82.50	44.87	3.35	50.70	14.00	32.01	96.68	34.00	60.40	12.50	73.70
		AO	5.10	81.90	42.73	3.30	51.03	14.40	32.22	94.34	35.00	63.10	15.40	100.00
		HLI	5.09	83.10	46.00	3.34	54.33	12.90	31.06	95.75	30.00	61.00	11.30	68.60
		HO	4.93	79.80	42.74	3.23	54.35	13.90	32.82	94.71	32.00	62.80	8.90	73.40
														
ORG	GULLIVER	ALI	5.69	83.30	45.02	3.38	37.81	15.90	39.21	85.37	35.00	57.80	10.50	73.70
		AO	4.70	81.80	42.28	3.27	39.82	15.50	37.13	76.15	36.00	58.40	6.50	68.20
		HLI	4.96	83.30	44.75	3.31	40.91	14.10	35.28	87.90	32.00	57.30	8.90	68.60
		HO	5.09	81.40	43.37	3.30	42.12	15.60	36.43	85.67	34.00	55.70	4.00	42.60
														
ORG	JULARO	ALI	5.67	82.20	44.97	3.38	47.27	14.10	33.31	96.57	37.00	59.20	9.50	81.20
		AO	4.54	80.60	43.45	3.37	47.71	15.00	30.78	91.92	37.00	62.80	8.40	100.00
		HLI	4.89	82.20	44.63	3.33	51.94	13.20	31.61	95.48	34.00	58.10	17.40	82.90
		HO	5.32	78.70	43.82	3.33	50.43	14.10	34.18	96.38	38.00	61.00	17.60	96.00
														
ORG	KARACHOW	ALI	4.56	79.60	38.25	3.13	48.62	17.40	50.94	39.88	28.00	60.20	4.20	59.80
		AO	3.75	78.90	37.49	3.13	48.87	16.60	48.07	41.31	25.00	60.30	3.90	53.00
		HLI	4.16	81.40	38.31	3.09	49.93	16.00	47.55	41.36	26.00	59.40	4.30	57.90
		HO	4.03	78.10	37.58	3.05	50.46	16.30	48.95	42.56	28.00	59.00	2.80	43.90
														
ORG	SANDOMIR	ALI	5.56	83.20	37.25	3.10	62.89	14.90	38.46	87.30^ab^	36.00	63.10	9.20	81.90
		AO	4.32	82.90	37.60	3.10	59.08	15.90	39.70	77.14^a^	36.00	67.70	9.20	79.00
		HLI	4.43	83.70	38.50	3.14	63.08	13.30	33.54	93.73^b^	31.00	62.40	16.80	81.60
		HO	4.75	81.30	36.54	3.05	62.51	14.00	35.41	88.10^ab^	34.00	60.60	4.40	48.80
														
ORG	SCARO	ALI	5.56	83.70	40.52	3.23	58.07	14.30	35.09	92.96	40.00	58.90	14.80	77.70
		AO	4.40	82.90	41.00	3.23	56.37	15.60	36.47	87.62	40.00	61.20	16.80	100.00
		HLI	4.64	84.30	41.89	3.24	56.68	14.50	34.77	93.23	39.00	60.60	11.70	86.40
		HO	4.84	80.90	40.32	3.22	59.07	14.40	35.20	94.91	40.00	61.20	15.70	76.00
														
ORG	WIWA	ALI	5.57	83.70	41.52	3.30	55.38	15.50	37.88	89.98	43.00	59.50	15.20	81.10
		AO	4.28	82.80	42.15	3.30	55.40	16.10	39.80	84.39	41.00	62.50	16.80	96.00
		HLI	4.19	83.90	40.97	3.26	56.75	14.90	36.60	92.63	42.00	62.30	12.60	87.40
		HO	4.74	40.40	40.76	3.25	57.00	15.50	39.52	90.61	43.00	63.00	16.70	87.20
														
CONV	ARNOLD	ALI	5.87	85.10	41.84	3.28	56.05	15.50	38.41	97.39	42.00	63.70	15.9*	97.20
		AO	4.80	85.00	39.50	3.20	54.61	16.00	36.73	97.58	42.00	64.80	6.60	100.00
		HLI	4.51	85.00	43.04	3.30	60.04	15.20	37.43	96.61	39.00	67.00	8.20	100.00
		HO	5.15	83.40	39.75	3.20	60.08	15.10	37.03	96.20	41.00	63.60	17.00	100.00
														
CONV	CAPO	ALI	6.25	84.30	40.75	3.20	57.83	14.20	34.36	97.49^b^	36.00	63.40	13.80	90.00
		AO	4.97	83.30	40.25	3.20	54.95	15.60	37.97	87.57^a^	35.00	66.20	12.70	94.30
		HLI	4.83	84.20	42.33	3.23	61.34	13.80	34.84	94.74^ab^	32.00	63.20	12.80	80.80
		HO	4.93	82.50	40.07	3.18	60.93	14.00	34.99	94.61^ab^	31.00	61.20	5.20	55.90
														
CONV	CLARO	ALI	5.57	81.90	42.23	3.33	60.65	14.40	36.70	91.97	34.00	63.40	9.90	76.60
		AO	4.64	82.10	41.76	3.30	57.93	14.70	37.17	84.55	34.00	66.30	8.00	79.80
		HLI	4.99	83.00	40.48	3.23	60.98	14.30	36.86	88.58	32.00	64.90	9.30	78.30
		HO	5.52	79.80	39.95	3.22	65.26	14.70	38.16	85.06	33.00	65.80	5.80	60.80
														
CONV	CH111-14426	ALI	5.77	79.20	41.23	3.35	67.11	14.80	34.81	94.78	33.00	62.60	10.80	78.90
		AO	4.81	79.30	44.17	3.40	64.75	15.80	36.73	90.35	32.00	63.30	16.50	100.00
		HLI	5.08	80.90	42.85	3.36	66.51	13.80	32.11	94.63	29.00	65.10	10.00	82.90
		HO	5.51	77.30	41.12	3.30	70.94	15.20	36.69	93.28	33.00	63.70	12.40	74.00
														
CONV	CH111-14631	ALI	5.67	82.60	41.21	3.33	58.80	13.70	32.35	98.60	39.00	59.20	13.40	88.90
		AO	4.59	80.30	40.50	3.27	53.59	14.30	32.15	90.19	34.00	62.30	11.70	100.00
		HLI	4.83	83.20	44.23	3.33	54.99	13.50	33.17	97.28	40.00	62.30	11.50	88.90
		HO	5.09	80.20	42.17	3.28	60.69	13.50	32.09	98.64	37.00	60.80	17.30	86.00
														
CONV	CH111-14663	ALI	5.84	81.70	35.86	3.18	63.50	14.40	34.04	95.31	34.00	59.80	7.10	62.20
		AO	4.70	81.40	34.70	3.13	57.58	15.20	34.70	89.57	33.00	61.20	9.80	76.00
		HLI	4.70	82.50	35.83	3.09	64.81	14.20	34.84	94.03	34.00	61.50	9.50	77.90
		HO	5.35	79.10	35.03	3.10	66.88	14.70	35.76	91.91	35.00	61.20	6.30	64.00
														
CONV	FLAMENKO	ALI	5.78	75.20	37.59^a^	3.10	46.20	13.30	27.28	98.77	30.00	58.20	8.30	61.00
		AO	4.92	73.60	35.82^a^	3.00	49.20	13.40	29.52	94.72	32.00	60.70	15.40	79.40
		HLI	4.91	77.90	42.43^b^	3.19	47.12	12.10	27.00	97.04	29.00	59.50	8.10	65.40
		HO	5.55	74.50	38.43^ab^	3.07	47.69	12.30	26.03	97.48	30.00	55.60	2.00	30.40
														
CONV	FOLKLOR	ALI	6.04	79.00^b^	38.31	3.18	55.86	13.10	27.64	98.50	34.00	59.60	6.30	70.10
		AO	5.02	78.00^b^	36.14	3.07	54.19	13.90	29.62	97.86	37.00	60.30	9.10	100.00
		HLI	5.68	77.50^b^	39.82	3.24	57.88	11.30	23.49	97.68	27.00	57.30	2.70	48.70
		HO	5.35	71.90^a^	34.46	3.08	61.73	12.30	25.70	97.65	31.00	55.30	1.40	32.70
														
CONV	LORENZO	ALI	4.90	78.70	38.73	3.30	60.35	16.00	39.56	97.75	46.00	63.30	10.00	88.00
		AO	4.55	79.60	38.66	3.27	57.81	15.80	36.95	96.20	45.00	64.00	15.50	100.00
		HLI	4.63	80.60	40.60	3.31	59.51	15.80	41.02	96.25	48.00	66.70	10.20	88.80
		HO	5.37	76.10	37.56	3.25	63.15	16.10	40.13	97.59	49.00	65.40	15.30	100.00
														
CONV	LUKULLUS	ALI	6.38^b^	83.00	43.14	3.28	50.01	14.70	34.69	98.22	41.00	61.30	11.10	91.70
		AO	5.20^ab^	83.00	43.68	3.23	47.74	15.10	33.50	96.49	39.00	62.80	10.00	100.00
		HLI	3.57^a^	82.90	42.26	3.26	49.86	13.80	32.47	98.07	37.00	64.80	13.90	96.00
		HO	5.02^ab^	80.40	40.97	3.15	54.62	13.90	33.22	97.84	37.00	58.20	8.50	68.50
														
CONV	MIDAS	ALI	6.57	83.00	41.94	3.33	49.48	13.90	32.61	97.73	35.00	60.40	13.80	86.60
		AO	5.44	82.20	40.56	3.27	49.00	14.20	31.43	94.41	35.00	60.30	18.00	100.00
		HLI	5.86	83.60	42.71	3.33	50.75	12.50	29.46	96.05	30.00	60.70	14.70	79.00
		HO	5.73	81.20	40.26	3.23	50.70	13.60	30.45	97.02	33.00	56.90	5.50	51.40
														
CONV	MONTDOR	ALI	5.53	81.37	43.76	3.30^a^	64.54	15.25	36.28	94.13	36.50	65.80	16.05	90.95
		AO	4.41	78.60	41.20	3.30^a^	62.66	15.13	32.88	97.84	39.00	66.30	7.30	100.00
		HLI	4.35	81.14	46.53	3.43^b^	60.99	15.41	37.26	95.90	48.14	69.10	11.70	100.00
		HO	4.98	78.18	44.48	3.37^ab^	62.29	15.31	35.31	96.58	41.58	68.10	12.60	91.90
														
CONV	MV BERES	ALI	5.34	80.50	47.16	3.38	44.57	16.50	42.33	66.33	30.00	67.70	5.40	71.40
		AO	4.64	78.30	42.30	3.23	50.44	15.40	36.37	71.23	29.00	68.90	6.60	93.00
		HLI	3.83	80.40	46.03	3.31	49.82	15.60	40.38	63.48	29.00	68.20	4.40	58.20
		HO	5.14	77.80	46.58	3.33	46.83	16.30	40.02	66.69	30.00	69.80	1.90	0.00
														
CONV	MV EMESE	ALI	5.38	84.50	49.44	3.40	39.86	14.70	35.35	95.15	33.00	63.20	13.80	89.30
		AO	4.83	82.70	46.84	3.30	45.41	14.20	32.22	95.79	33.00	64.70	9.90	100.00
		HLI	4.70	84.50	47.70	3.36	44.20	14.00	34.08	93.19	34.00	64.40	13.90	86.90
		HO	4.94	82.10	48.19	3.35	42.96	14.80	35.78	93.81	36.00	69.50	9.20	84.40
														
CONV	MV KOLO	ALI	5.64	81.60	42.75	3.25	50.14	15.00	36.21	94.85	38.00	63.30	8.70	82.10
		AO	4.97	80.70	39.58	3.13	50.49	14.80	35.43	91.04	34.00	64.90	11.30	96.00
		HLI	5.01	82.20	44.28	3.24	51.11	14.60	37.27	92.34	37.00	65.30	13.30	86.30
		HO	5.33	79.00	40.57	3.13	53.75	14.70	36.73	94.70	40.00	67.60	8.30	90.10
														
CONV	MV KOLOMPOS	ALI	5.95	78.50	48.61	3.38	50.80	14.70	35.21	72.37	28.00	61.10	7.90	69.10
		AO	4.83	77.50	43.16	3.23	52.53	14.00	32.93	63.35	27.00	59.60	9.00	73.40
		HLI	5.27	79.50	46.24	3.30	53.15	13.40	33.89	67.72	27.00	60.30	6.50	66.90
		HO	5.65	76.20	44.32	3.28	53.77	14.30	35.73	68.47	29.00	64.40	4.80	65.20
														
CONV	MV TALLER	ALI	6.40	82.70	44.46	3.28	53.04	13.60	32.73	83.78	29.00	65.00	11.10	69.90
		AO	5.16	80.50	38.98	3.07	55.95	13.80	32.18	76.21	29.00	65.00	11.30	75.00
		HLI	5.07	82.50	43.11	3.21	48.41	13.40	35.21	73.19	29.00	65.90	4.50	61.00
		HO	5.75	80.40	42.16	3.22	53.57	13.30	32.46	87.19	29.00	69.20	6.00	73.70
														
CONV	RENAN	ALI	5.73	80.00	44.34	3.23	51.90	14.40	31.69	98.47	34.00	62.50	10.10	90.50
		AO	4.79	79.00	42.43	3.13	53.35	14.20	30.02	98.28	31.00	60.80	14.50	100.00
		HLI	5.02	81.70	48.17	3.29	53.99	14.00	33.81	97.23	33.00	62.90	15.20	92.50
		HO	5.13	80.10	45.59	3.22	57.28	13.60	31.68	96.83	31.00	59.70	6.70	56.10
														
CONV	SURETTA	ALI	4.92	79.30	37.53	3.15	61.76	15.10	39.01	85.50	34.00	62.90	9.00	72.20
		AO	4.76	78.90	36.85	3.13	59.87	14.70	37.20	75.62	32.00	63.10	17.00	83.90
		HLI	4.78	80.40	39.20	3.19	64.08	13.90	36.46	87.23	31.00	65.20	9.40	77.60
		HO	5.55	76.50	37.87	3.15	63.63	14.20	36.69	85.40	31.00	63.40	4.20	59.00
														
CONV	TITLIS	ALI	5.33	81.90	42.23	3.30	50.75	14.70	37.18	92.71	38.00	62.50	11.50	74.40
		AO	4.53	81.30	42.18	3.30	50.78	15.30	36.57	90.94	39.00	65.20	15.00	100.00
		HLI	4.97	82.70	42.29	3.24	55.73	13.60	34.16	95.60	35.00	64.20	15.00	88.00
		HO	5.31	79.70	41.80	3.25	57.24	14.90	37.79	94.89	40.00	64.50	14.30	83.90
														
BFOA	BITOP	ALI	5.94	84.70	33.60	3.43	57.53	14.50	37.61	96.86	35.00	65.60	12.00	86.50
		AO	4.93	84.00	43.74	3.40	55.53	15.30	36.07	95.47	36.00	66.50	14.50	96.00
		HLI	5.00	84.40	44.03	3.33	62.51	14.40	36.36	96.79	34.00	67.40	13.70	88.00
		HO	5.18	83.40	43.44	3.32	63.26	13.60	34.22	96.41	32.00	64.30	13.00	76.70
														
BFOA	BLASIUS	ALI	6.00	82.00	37.97	3.23	59.44	15.00	37.39	96.95	44.00	63.10	10.30	82.60
		AO	5.21	81.80	38.55	3.20	61.54	15.90	38.70	88.97	43.00	65.10	15.50	100.00
		HLI	4.68	82.50	38.00	3.19	61.95	14.20	34.95	93.92	37.00	64.30	10.30	81.90
		HO	5.33	79.20	37.44	3.17	63.97	14.60	38.03	94.44	40.00	60.90	5.20	53.60
														
BFOA	HENDRIX	ALI	5.82	76.60	36.07	3.18	61.43	13.60	29.15	98.68^b^	35.00	57.90	16.80	88.60
		AO	4.79	77.40	36.09	3.10	59.74	12.60	30.25	94.97^a^	32.00	57.40	17.70	100.00
		HLI	5.34	82.20	38.75	3.11	57.46	12.20	26.89	98.23^b^	33.00	59.80	17.70	94.00
		HO	4.86	77.60	35.86	3.03	57.69	14.20	29.19	98.41^b^	35.00	61.60	13.30	81.00
														
BFOA	PEPPINO	ALI	6.25	83.10	38.37	3.23	60.05	14.90	35.85	98.22	39.00	62.50	10.50	88.90
		AO	4.98	82.10	36.28	3.17	60.97	15.50	35.40	96.39	36.00	64.00	7.30	100.00
		HLI	4.74	82.80	39.40	3.23	63.82	14.40	35.04	94.84	35.00	64.70	11.80	87.90
		HO	5.05	80.20	36.95	3.13	64.38	14.10	33.95	96.72	35.00	61.00	11.30	74.40
														
BFOA	PIRENEO	ALI	6.16	84.00	43.52	3.38	59.46	15.10	37.36	97.65	33.00	62.00	9.80	86.60
		AO	5.07	83.80	42.38	3.27	55.11	15.30	33.53	97.17	38.00	61.80	12.30	100.00
		HLI	5.16	83.50	42.31	3.27	58.21	13.70	32.86	97.68	32.00	61.80	12.60	87.00
		HO	5.47	82.00	40.58	3.18	63.08	14.70	36.18	97.68	37.00	60.30	14.40	81.50
														
BFOA	SKERZZO	ALI	6.07	80.60	38.36	3.10	49.58	14.50	36.08	98.25^b^	35.00	59.60	7.20	69.00
		AO	5.01	80.40	38.61	3.10	46.91	14.10	33.75	89.27^a^	37.00	59.60	8.80	71.30
		HLI	5.39	82.20	40.75	3.11	49.73	12.70	31.79	95.16^ab^	31.00	60.20	6.10	61.50
		HO	5.21	79.90	36.50	2.98	54.35	13.60	33.48	94.89^ab^	34.00	57.50	6.30	54.20
														
BFOA	STEFANUS	ALI	6.08	86.00	42.35	3.30	58.26	14.70	36.73	98.03	36.00	64.50	13.50	93.60
		AO	4.95	84.40	39.67	3.13	53.65	15.40	36.03	94.00	37.00	65.50	13.40	91.90
		HLI	5.12	85.40	42.29	3.21	60.29	13.60	33.06	96.92	32.00	64.40	15.40	92.50
		HO	5.62	84.00	40.02	3.13	62.92	13.70	32.12	94.89	33.00	63.10	11.10	74.00
														
BFOA	TOBIAS	ALI	6.02^b^	83.50	40.58	3.20	55.85	15.90	40.04	93.62	37.00	63.60	8.70	83.00
		AO	4.71^ab^	83.00	39.85	3.17	54.49	16.40	39.73	85.81	37.00	64.30	10.20	93.00
		HLI	3.53^a^	83.20	41.11	3.23	60.12	15.20	39.60	90.05	33.00	63.90	13.70	96.00
		HO	4.94^ab^	83.20	41.53	3.20	59.47	14.90	37.58	92.47	33.00	59.10	4.20	45.60
														
	Grand total		Yield	TW	TKW	KW	HI	Protein	Gluten	GI	Zeleny	Wabs	Stability	QN

	Mean		5.09	81.00	41.19	3.23	56.43	14.40	35.39	89.91	35.00	62.80	10.60	79.50
	*N*		1332	737	736	737	699	734	734	734	734	219	219	219
	Std. dev		1.61	6.30	5.03	0.15	8.74	2.70	7.34	13.35	8.00	3.40	4.50	17.70
														
		GS	Yield	TW	TKW	KW	HI	Protein	Gluten	GI	Zeleny	Wabs	Stability	QN

	Mean of sites	ALI	5.72^c^	81.99^b^	41.34^b^	3.26^b^	53.46^a^	15.25^a^	37.19^a^	90.78^c^	35.57^ab^	62.14^a^	10.69^ab^	79.83^b^
		AO	4.73^a^	79.68^ab^	40.16^ab^	3.21^a^	50.92^a^	16.33^bc^	37.57^a^	85.98^ab^	37.64^b^	63.25^b^	11.61^ab^	90.69^c^
		HLI	4.77^a^	81.80^b^	37.97^a^	3.25^b^	58.35^b^	17.10^c^	42.11^b^	87.65^b^	42.19^c^	87.64^c^	12.09^b^	87.18^bc^
		HO	5.15^b^	77.40^a^	38.99^ab^	3.19^a^	57.24^b^	15.64^ab^	37.18^a^	85.29^a^	34.03^a^	62.59^ab^	9.07^a^	67.50^a^

Homogeneous subset indicators (a–d) based on Tukey׳s post hoc test at the *p*=0.05 significance level are also shown separately for each trait.

Values with no letter in the mean columns indicate, that sites do not significantly different (*p*<0.05) from each other. Significant differences are marked with different letters.

**Table 4:** Mean values and standard deviations (SD) of the physical, grain compositional and breadmaking quality traits (low input and organic sites in Austria and Hungary, 2011–2013) assessed for 37 winter wheat varieties with three different breeding origins.

Instruments	Traits	LI								O							
		TOTAL		CONV		BFOA		ORG		TOTAL		CONV		BFOA		ORG	
		Mean	SD	Mean	SD	Mean	SD	Mean	SD	Mean	SD	Mean	SD	Mean	SD	Mean	SD
FOSS Tecator 1241	Test weight (kg/hl)	81.22***	7.62	81.60	3.15	82.98	3.20	83.12	2.38	78.95***	10.22	79.30	3.82	81.55	3.37	77.71	15.70
	Protein content (%)	14.86	2.65	14.95	2.39	14.85	2.37	15.49	2.52	15.33	2.43	15.24	2.16	15.14	2.01	16.04	2.42
	Starch content (%)	56.91**	5.34	57.34	2.15	57.41	2.49	57.33	2.57	56.42**	4.70	56.81	1.91	56.75	2.14	56.59	2.35
	Gluten content (%)	34.74	6.71	34.90	6.14	34.89	6.48	37.36	6.37	36.11	5.75	35.59	5.02	35.95	5.20	38.21	5.43
	Water absorption (%)	68.55	10.20	68.84	8.77	70.00	8.98	71.41	9.64	68.17	8.74	67.83	7.16	68.76	7.55	69.96	7.66
	Zeleny sedimentation (ml)	49.44	13.95	49.60	13.59	50.93	14.44	54.99	12.00	52.48	12.84	51.09	12.80	52.26	12.09	56.84	11.46
Perten Falling Number system 1500	Falling number (s)	386.99	88.26	389.09	84.01	389.77	73.81	396.23	83.00	374.16	120.68	369.80	119.98	383.99	114.88	381.60	124.11
Kjeltec 1035 Analyzer	Protein wholemeal (%)	14.07	3.00	14.15	2.80	14.13	2.81	14.65	2.94	14.59	2.71	14.48	2.48	14.48	2.45	15.24	2.75
Chopin CD1	Flour yield (%)	51.27	5.64	51.56	3.71	51.37	3.68	54.27	4.55	52.51	5.36	52.48	3.40	52.15	3.25	54.13	4.69
Glutomatic 2200	Gluten content (%)	34.43	8.03	34.50	7.64	34.63	7.61	37.65	8.17	35.33	7.01	34.47	6.16	34.77	5.71	38.50	7.66
	Gluten spread (mm)	4.69	2.90	4.67	2.98	4.30	2.13	6.94	5.09	5.12	3.47	4.64	3.03	4.28	2.01	7.04	4.65
	Gluten Index	91.19***	12.91	91.39	11.07	96.17	4.06	83.04	19.08	88.90***	14.64	90.72	11.48	94.74	4.83	81.55	18.10
SediCom System	Zeleny sedimentation (ml)	34.01	8.78	34.35	8.62	34.55	8.16	34.78	9.45	35.03	7.09	34.74	6.56	35.66	5.68	35.86	8.02
Marvin System	Thousand-kernel weight (g)	42.18***	5.84	42.70	5.01	40.09	6.08	41.45	5.15	40.18***	5.23	40.86	4.55	39.20	4.13	40.42	4.36
	kernel size % (<2.50 mm)	1.96	1.85	1.83	1.84	2.51	1.94	1.83	2.00	2.94	2.85	2.82	2.56	3.65	3.43	2.55	2.92
	% (2.50–2.75 mm)	6.67	4.49	6.41	4.49	7.98	5.39	7.10	5.56	8.97	5.77	8.61	5.29	10.56	6.43	8.42	6.11
	% (2.75–3.00 mm)	15.54	7.45	15.19	7.44	16.92	8.10	16.37	8.22	18.14	7.00	17.65	6.91	19.75	7.21	18.09	6.85
	% (3.00–3.25 mm)	24.75	6.48	24.37	6.27	26.13	6.15	25.67	5.92	25.90	5.09	25.70	4.76	25.89	4.65	26.89	5.18
	% (3.25–3.50 mm)	24.62	5.72	25.05	5.38	23.55	6.17	24.90	6.06	22.76	5.90	23.18	5.55	21.77	6.41	23.13	5.67
	% (3.50–3.75 mm)	16.78	7.64	17.35	7.62	15.45	8.33	15.71	8.23	14.03	6.83	14.53	6.47	13.03	7.52	13.99	7.05
	% (3.75–4.00 mm)	7.03	5.02	7.44	5.10	5.82	5.26	6.81	5.57	5.42	4.13	5.79	4.21	4.42	3.78	5.50	4.24
	% (4.00–4.25 mm)	1.84	2.04	1.97	2.12	1.37	1.63	1.34	1.61	1.23	1.41	1.44	1.55	0.76	0.90	1.18	1.37
	% (4.25–4.50 mm)	0.30	0.57	0.34	0.60	0.23	0.54	0.26	0.49	0.20	0.37	0.23	0.41	0.13	0.27	0.18	0.33
	% (>4.74)	0.05	0.17	0.05	0.18	0.04	0.12	0.00	0.04	0.03	0.10	0.03	0.10	0.03	0.10	0.03	0.10
	Kernel width (mm)	3.24***	0.31	3.27	0.15	3.23	0.16	3.24	0.16	3.18***	0.28	3.22	0.14	3.16	0.15	3.21	0.15
	Kernel length (mm)	6.50	0.63	6.55	0.31	6.38	0.20	6.37	0.26	6.44	0.56	6.57	0.32	6.37	0.18	6.37	0.27
Perten SKCS 4100	Thousand kernel weight (g)	41.08	5.57	41.66	4.48	39.45	3.91	40.71	4.82	39.24	4.74	39.84	3.87	38.56	3.51	39.36	4.10
	Kernel diameter (mm)	2.61	0.28	2.64	0.16	2.55	0.14	2.61	0.17	2.55	0.24	2.58	0.15	2.51	0.14	2.58	0.17
	Hardness index	54.96	9.51	55.32	8.72	58.62	7.04	55.24	9.68	56.61	9.48	56.51	8.36	59.42	7.00	55.53	10.46
Brabender Farinograph	Water absorption (%)	61.73	9.22	63.09	2.98	62.77	2.70	61.62	3.89	61.51	10.18	63.37	3.62	62.00	2.81	63.30	4.58
	Dough development time (min)	9.16*	5.28	9.04	5.42	11.64	5.21	7.86	4.97	10.01*	5.92	10.35	6.42	10.33	5.90	9.38	5.57
	Dough stability (min)	10.31***	4.37	10.42	4.44	11.80	3.72	10.81	5.13	10.12***	4.62	10.00	4.65	11.16	3.89	10.06	5.36
	Dough softening at 10 min (FU)	194.05*	219.25	184.74	221.62	254.74	240.91	126.67	192.31	235.54*	221.60	251.95	231.46	236.88	231.06	197.39	224.09
	Farinograph quality number	85.54***	20.37	88.44	14.35	94.63	10.28	84.19	12.93	77.43***	22.49	79.47	23.09	80.83	18.00	75.91	19.40

LI: low input, O: organic, TOTAL: including all varieties, CONV: conventionally bred varieties, BFOA: varieties developed by combined breeding, ORG: organically bred varieties.

* Significant differences at the 0.05 probability levels, respectively.

** Significant differences at the 0.01 probability levels, respectively.

*** Significant differences at the 0.001 probability levels, respectively.

**Table 5:** Most stable varieties at organic (O) and low input (LI) sites, with above-average mean values for the trait but the lowest coefficient of variation (mean;CV). The varieties were developed by the following breeding methods: CONV: conventional, ORG: organic, BFOA: combination of methods. CV values in brackets.

**Management**	**LI**	**O**	**LI**	**O**	**LI**	**O**
Traits	**ORG**	**ORG**	**BFOA**	**BFOA**	**CONV**	**CONV**
Yield (t/ha)	Sandomir (5.0;28.9)	Gulliver (4.9;18.9)	Bitop (5.5;31.6)	Skerzzo (5.1;21.1)	Capo (5.5;31.9)	Mv Kolo (5.2;21.1)
Test weight (kg/hl)	Scaro (84.1;2.5)	Scaro (81.6;3.1)	Stefanus (85.6;2.5)	Stefanus (84.1;2.5)	Mv Emese (84.5;2.5)	Capo (82.8;2.7)
Thousand kernel weight (g)	Donnato (45.6;8.4)	Scaro (40.6;4.5)	Stefanus (42.3;8.3)	Bitop (43.5;5.6)	Mv Taller (43.6;6.8)	Mv Emese (47.7;4.7)
Kernel width (mm)	Donnato (3.4;3.9)	Scaro (3.2;1.4)	Stefanus (3.2;3.6)	Bitop (3.3;3.0)	Mv Beres (3.34;3.6)	Montdor (3.3;1.6)
Flour yield (%)	Karachow (58.7;1.9)	Karachow (58.5;2.2)	Skerzzo (54.1;2.6)	Skerzzo (54.7;2.7)	Lukullus (55.6;2.8)	Flamenco (54.0;3.2)
Falling number (s)	Aszita (397.8;10.6)	Wiwa (420.9;14.7)	Hendrix (377.1;9.9)	Stefanus (432.0;16.2)	Mv Kolompos (439.9;12.2)	CH111-14426 (409.9;20)
Protein wm (%)	Karachow (16.5;13.9)	Gulliver (15.5;14.0)	Bitop (14.4;14.9)	Pireneo (14.9;15.5)	Mv Emese (14.3;15.5)	Lorenzo (15.9;14.7)
Gluten (%)	Karachow (48.8;9.9)	Karachow (48.7;11.9)	Bitop (36.8;16.6)	Tobias (38.3;15.9)	Mv Emese (34.5;15.9)	Lorenzo (39.1;11.5)
Gluten index	Donnato (96.1;2.4)	Donnato (94.6;3.9)	Hendrix (98.4;1.2)	Pireneo (97.5;1.8)	Lukullus (98.1;0.9)	Folklor (97.7;1.1)
Zeleny sedimentation (ml)	Jularo (35.1;18.6)	Wiwa (42.2;13.5)	Bitop (34.7;16.9)	Pireneo (37.2;9.0)	Lorenzo (47.3;17.8)	Lorenzo (47.8;9.2)
Water absorption (%)	Sandomir (62.7;1.1)	Butaro (72.2;0.9)	Bitop (66.5;1.7)	Bitop (65.4;2.4)	Mv Beres (67.9;1.5)	CH111-14426 (63.5;0.5)
Dough stability (min)	Butaro (13.6;8.5)	Wiwa (16.7;0.4)	Hendrix (17.2;8.9)	Bitop (13.7;7.7)	Lorenzo (10.1;10.7)	Lorenzo (15.4;0.9)
Dough softening (FU)	Karachow (58.2;39.8)	Aszita (28.0;55.6)	Skerzzo (45.3;37.3)	Skerzzo (49.0;66.4)	Mv Kolompos (32.5;56.2)	Mv Taller (22.0;12.8)

In the case of dough softening, the lowest mean values were taken into consideration. Wm: wholemeal.
